# Knot_pull—python package for biopolymer smoothing and knot detection

**DOI:** 10.1093/bioinformatics/btz644

**Published:** 2019-08-16

**Authors:** Aleksandra I Jarmolinska, Anna Gambin, Joanna I Sulkowska

**Affiliations:** Centre of New Technologies, Warsaw 02-097, Poland; Department of Mathematics, Informatics and Mechanics, University of Warsaw, Warsaw 02-097, Poland; Department of Mathematics, Informatics and Mechanics, University of Warsaw, Warsaw 02-097, Poland; Centre of New Technologies, Warsaw 02-097, Poland; Faculty of Chemistry, University of Warsaw, Warsaw 02-093, Poland

## Abstract

**Summary:**

The biggest hurdle in studying topology in biopolymers is the steep learning curve for actually seeing the knots in structure visualization. Knot_pull is a command line utility designed to simplify this process—it presents the user with a smoothing trajectory for provided structures (any number and length of protein, RNA or chromatin chains in PDB, CIF or XYZ format), and calculates the knot type (including presence of any links, and slipknots when a subchain is specified).

**Availability and implementation:**

Knot_pull works under Python >=2.7 and is system independent. Source code and documentation are available at http://github.com/dzarmola/knot_pull under GNU GPL license and include also a wrapper script for PyMOL for easier visualization. Examples of smoothing trajectories can be found at: https://www.youtube.com/watch?v=IzSGDfc1vAY.

**Supplementary information:**

Supplementary data are available at *Bioinformatics* online.

## 1 Introduction

The last decade has seen a large increase in the number of studies related to the protein topology (some of recent discoveries are reviewed in Jarmolinska *et al.*, 2019). While first structures with non-trivial folds have been described much earlier, only recently databases of such folds were created. Currently, there are over 1500 knotted or slipknotted protein chains described in KnotProt 2.0 (Dabrowski-Tumanski *et al.*, 2019), and almost 10 000 protein links in LinkProt (Dabrowski-Tumanski *et al.*, 2016). Screening of available RNA structures has found entanglements, potentially as a result of low resolution (Micheletti *et al.*, 2015). Recent advances in the study of chromatin structure gave rise to new 3D models—many of which contain entanglements, including composite knots, as of yet unknown in proteins (Sulkowska *et al.*, 2018). Still, the subject of molecular entanglements remains relatively unknown to a lot of researchers, including those studying protein structures. One obvious reason is the steep learning curve for actually seeing the knots in a 3D structure visualization.

Knot_pull is a tool designed to allow an easy analysis of topological intricacies of a structure, by providing the user with a trajectory of smoothing steps—from the full structure, to the minimal number of coordinates preserving the original topology (with regard to fixed position of chain termini)—as well as the knot type (including separation of composite knots, and indication of any linking present).

Knot_pull has been successfully used for protein and RNA structures, as well as chromatin models, and due to universal input formats can be used for any open polymer described by a sequence of 3D coordinates.

## 2 Materials and methods

Knot_pull smooths and simplifies the provided structure in a topologically conscious way—that is without allowing the implicit ‘chain’ to pass through itself. The backbone of the chain is described by a sequence of 3D coordinates called beads. For each chain, all the other chains provided are seen as obstacles in the smoothing process. When working on a single chain, it is possible to specify the positions of a subchain to be smoothed. This allows analysis of slipknots—entanglements where the full chain is trivial, but removing a number of beads from the ends results in a knotted structure. Additionally, when only a single chain is analyzed, the smoothing is done in an orientation-independent way, which, to the best of our knowledge, makes knot_pull the first such algorithm.

### 2.1 Smoothing algorithm and knot detection

**Fig. 1.**
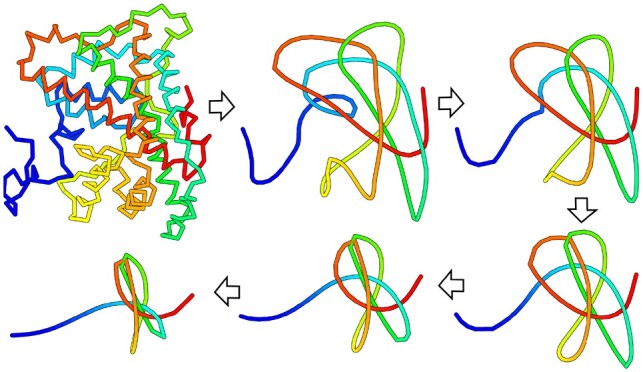
Some of the smoothing steps for the structure with RCSB PDB Id 3bjx, chain A, which contains a knot with 6 crossings when projected on a plane (6_1_ knot)

Smoothing proceeds in a manner similar to the one discussed in (Taylor, 2000), with some modifications to ensure the deterministic nature of the results (see Supplementary Algorithm S1 for the orientation-independent method, and Supplementary Algorithm S2 for the one-direction approach). An exemplary smoothing trajectory is shown in Figure 1. The triangle crossing condition described in (Koniaris and Muthukumar, 1991) ensures that the topology of the chain is not changed. After no more changes can be made (or the current configuration was already visited), all superfluous beads are removed, leaving the minimal topologically necessary number of points (i.e. trivial chain usually simplifies to just two original end points). All data is stored as list of coordinates, and as such the space complexity is linear to the length of the molecule. Time complexity depends on how densely packed is the structure, so it cannot be easily estimated (see Supplementary Fig. S1).

If the structure is a composite knot, subentanglements are found—as non-trivial subchains that cannot be smoothed even if separated from the rest of the structure (see Supplementary Algorithm S2). When at least two chains are present in the analysis, their linking is checked in an analogous manner. Particular knot type of each (sub)entanglement is found by considering its Dowker-Thistlewaithe (DT) code (Dowker and Thistlethwaite, 1983), which is then simplified through a series of Reidemeister moves-based calculations (Reidemeister, 1927; described in the SI) until a minimal code is obtained and corresponding Alexander-Briggs (AB) notation (Alexander and Briggs, 1926; Hoste *et al.*, 1998) found.

In DT code the crossings are numbered following the chain, thus an open chain is implicitly connected in a way that did not introduce any new crossing. Additionally, as proteins have a well-defined beginning and direction (going from the N to the C terminus) two different realizations of a knot of the same type can be differentiated, up to small differences due to the planar projection, similar to KnotProt 2.0’s knotoid features.

The smoothing method used, which ‘tightens’ the entanglement while maintaining the terminal beads positions, improves the protrusion of the ends from the structure Taylor, 2000. This ensures that a ‘proper’ closing of a chain—one that doesn’t introduce new crossings—exists, so can be implicitly added through the DT code. The idea of studying the topology of an open polymer, in a planar projection and without explicitly closing it, is similar to the knotoid concept [introduced for biomolecules in (Goundaroulis *et al.*, 2017)]. The main difference is that the proper DT code implies a connection between first and last crossing (the segment ‘missing’ in open chains). Thus, if the code is syntactically correct, it describes a three dimensional closed curve. This approach, combined with the deterministic smoothing algorithm, makes knot_pull unique amongst the popular knot finding algorithms. Most use knot invariants defined only for closed curves (Lickorish, 2012), and to get a result reasonably independent from the choice of a closing path, the calculations are repeated for many random closures—and give only a *probability* of a given knot type, whereas knot_pull’s topology results are fully deterministic for a given structure.

### 2.2 Usage

Package provides a command line tool called *knot*_*pull*_*check* for smoothing 3D chains and determining their topology.

Input structures can be provided either using an RCSB PDB Id, or as a local, PDB, CIF or XYZ formatted, file. It is possible to specify the atom name to be used for backbone trace. Multiple chains can be selected for the analysis (this allows for finding interchain links), by default all chains present will be used. There is no limit on the length of the chain, nor the number of chains. For XYZ file format chains should by separated by an ‘END’ keyword (always all chains present will be analyzed).

With default settings, only the smoothing is performed, and the resulting structure (only selected backbone atoms) in PDB format is outputted to standard output (an output file can also be specified). A full trajectory of consecutive smoothing steps can also be returned. When a ‘-k’ option is specified, a topological analysis of the final smoothed structure is performed and both AB notation and simplified DT code are outputted.

Additionally, a wrapper for PyMOL Molecular Graphics System (*knot*_*pull*_*show*) is included, for user-friendly visualization of the smoothing trajectories.

### 2.3 Validation

Knot_pull was tested against the KnotProt 2.0 database, which is hand curated. All the chains annotated in the database as knotted (1054 in total) have been found to return the same knot type for the full chain (disregarding the chirality, as it is not included in knot_pull’s AB tabulation). For 503 slipknotted chains there were 3 cases where knot_pull reported a knot instead of the (expected) unknot—all three structures actually contain an (albeit very shallow) knot. Additionally, over 94% of those structures were reduced to only 2 points during smoothing. From chains reported by KnotProt 2.0 as trivial, 65 000 were randomly selected and tested, and all of them reduced to 2 points after smoothing, except for seven structures which actually contain knots (Supplementary Table S1), and one which by homology should be knotted, but was calculated as an unknot due to a gap in the structure (PDB Id 4kjs chain A). Testing available RNA structures confirmed results of (Micheletti *et al.*, 2015), with only the structures reported there exhibiting non-trivial topology.

The orientation-independent algorithm was tested on the coordinates proposed in (Millett *et al.*, 2005) (shown, for previous methods, to produce different topology depending on the orientation of the chain), and resulted in an identical smoothing trajectory for the reversed chain.

Other publicly available software for entanglement, with standalone (non-webserver) versions, are PyKnot (Lua, 2012) and KymoKnot (Tubiana *et al.*, 2018), which use the polynomial invariants for knot type determination, and as such are susceptible to errors caused by wrong curve closure (in case of KymoKnot this is mitigated by the Minimally-Interfering closure scheme).

## Supplementary Material

btz644_Supplementary_Data
