# Supplementary material for: Knot_pull—python package for biopolymer smoothing and knot detection
Source: Bioinformatics. 2019 Aug 16;36(3):953–5. doi: 10.1093/bioinformatics/btz644 (PMC9883683; doi:10.1093/bioinformatics/btz644)
Supplement: btz644_Supplementary_Data [file bioinformatics_36_3_953_s2.pdf]

Supplement

1 Smoothing algorithm

The backbone of the chain(s) is described by a sequence of 3D coordinates called beads. Gaps in the structure, defined as distance of over 4Å between consecutive beads, are filled in with a straight line (made up of new beads). Smoothing algorithm is described in Algorithm 1 (the orientation-independent method, which proceeds symmetrically from both ends) and Algorithm 2 (oriented smoothing – starting from the N terminus of the first chain in the analysis; used when more than one chain is present). Both use triangle crossing condition described by (Koniaris and Muthukumar, 1991). Links between chains, and entanglements within a chain that can be separated by splitting it into sub-chains are found using Algorithm 3.

2 Dowker-Thistlewaithe code simplification

Full Dowker-Thistlethwaithe (DT) code of a 3D structure is found by projecting it on a plane (structure is rotated along X, Y and Z axes to get a formally valid code). Then, by following the chain starting from the first bead (N terminus in proteins), each intersection of two bead-connecting segments (crossing) is sequentially numbered as it is encountered. This gives two values – one of which is marked to indicate which was added while going over the crossing – $C(a, b)$. A sequence of those crossing values forms the DT code. It has been proven that the code is only valid if every crossing is annotated with one even and one odd number (Dowker and Thistlethwaite, 1983).

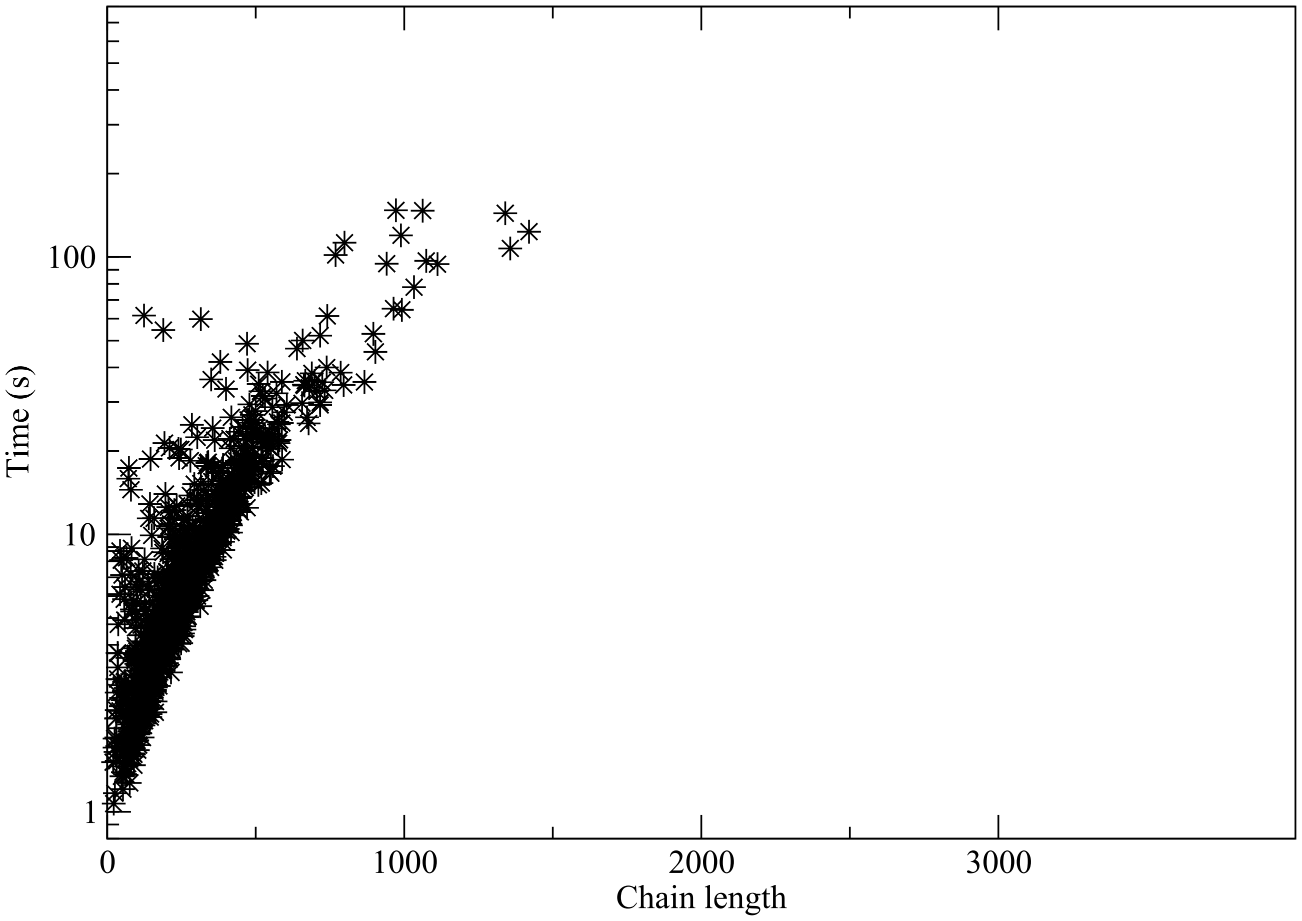

Fig. 1. Time complexity of the smoothing process using the oriented Algorithm for a single chain is roughly exponential to its length.

Resulting code is then simplified through a series of syntactic modifications based on the three Reidemeister moves of the chain (which have been shown to be sufficient to relate knot diagrams with the same knot type (Reidemeister, 1927)):

1. (untwisting):
   - a simple loop (a crossing annotated with two consecutive values) can be removed with no influence on the rest of the structure (Figure 2A)
   - when another segment passes through a simple loop (assuming $C.a$ and $C.b$ are values in a DT annotation of a crossing, and $C.a \neq C.b$: $abs(C_1.a - C_1.b) = 2; C_1.a < C_2.a, C_3.a < C_1.b$ and the chain goes sequentially through C_1 and C_2 on the same side (over/under), different than through C_3) the twisted crossing can be removed but the order of "internal" crossings along the strike-through segment must be reversed (Figure 2C);

Algorithm 1 Orientation-independent smoothing algorithm for a continuous sequence of 3D coordinates

```
INPUT: a doubly linked list of beads atoms
OUTPUT: a shorter doubly linked list of beads
segment ≡ virtual line connecting two consecutive beads
cross ≡ strike through the surface spanned between given points

function symmetric_smooth (atoms)
while bead removed, added or moved by > 0.05Å do
  Ntriangle[left,middle,right] ← first three beads in atoms
  Ctriangle[left,middle,right] ← last three beads in atoms
  while Ntriangle and Ctriangle didn't bypass each other do
    if Ntriangle fully overlaps Ctriangle then
      if other segment crosses Ntriangle then
        if distance(Ntriangle.left,Ntriangle.middle) > 4 Å then
          add bead between Ntriangle.left and Ntriangle.middle at avg(Ntriangle.left,Ntriangle.middle)
        if distance(Ntriangle.middle,Ntriangle.right) > 4 Å then
          add bead between Ntriangle.right and Ntriangle.middle at avg(Ntriangle.right,Ntriangle.middle)
        break
      else
        if distance(Ntriangle.left,Ntriangle.right) < 4 Å then
          remove Ntriangle.middle from chain
          expand Ntriangle to three beads, relative to the most N-terminal current one
        else
          move Ntriangle.middle to avg(Ntriangle.left,Ntriangle.right)
        break
    else
      if surfaces spanned on Ntriangle and Ctriangle cross each other then
        move Ntriangle by one towards C terminus
        move Ctriangle by one towards N terminus
      else
        Nobstacles ← bool(there are segments crossing Ctriangle)
        Cobstacles ← bool(there are segments crossing Ctriangle)
        if Nobstacles then
          if Cobstacles then
            move Ntriangle by one towards C terminus
            move Ctriangle by one towards N terminus
          else
            if distance(Ctriangle.left,Ctriangle.right) < 4 Å then
              remove Ctriangle.middle from atoms
              expand Ctriangle to three beads, relative to the most C-terminal current one
              move Ntriangle by one towards C terminus
            else
              move Ctriangle.middle to avg(Ctriangle.left,Ctriangle.right)
              move Ntriangle by one towards C terminus
        else
          if Cobstacles then
            if distance(Ntriangle.left,Ntriangle.right) < 4 Å then
              remove Ntriangle.middle from atoms
              expand Ntriangle to three beads, relative to the most N-terminal current one
              move Ctriangle by one towards N terminus
            else
              move Ntriangle.middle to avg(Ntriangle.left,Ntriangle.right)
              move Ctriangle by one towards N terminus
          else
            if Ntriangle and Ctriangle overlap on two beads then
              break
            else
              if distance(Ctriangle.left,Ctriangle.right) < 4 Å then
                remove Ctriangle.middle from atoms
                expand Ctriangle to three beads, relative to the most C-terminal current one
              else
                move Ctriangle.middle to avg(Ctriangle.left,Ctriangle.right)
              if distance(Ntriangle.left,Ntriangle.right) < 4 Å then
                remove Ntriangle.middle from atoms
                expand Ntriangle to three beads, relative to the most N-terminal current one
              else
                move Ntriangle.middle to avg(Ntriangle.left,Ntriangle.right)
```

Algorithm 2 Smoothing algorithm for an oriented sequence of 3D coordinates

INPUT: a doubly linked list of beads *atoms*
OUTPUT: a shorter doubly linked list of beads
segment ≡ virtual line connecting two consecutive beads
cross ≡ strike through the surface spanned between given points

```
function smooth (atoms)
while bead removed, added or moved by > 0.05Å do
    Ntriangle[left,middle,right] ← first three beads in atoms
    while Ntriangle has three beads do
        if less than two beads remain till end of current chain then
            move Ntriangle by one towards C terminus
        if no segment crosses Ntriangle then
            if distance(Ctriangle.left,Ctriangle.right) > 4 Å then
                move Ctriangle.middle to avg(Ctriangle.left,Ctriangle.right)
                move Ntriangle by one towards C terminus
            else
                move Ntriangle.middle from atoms
        else
            if distance(Ctriangle.left,Ctriangle.right) > 4 Å then
                add bead between Ntriangle.left and Ntriangle.middle at avg(Ntriangle.left,Ntriangle.middle);
                move Ntriangle by one towards C terminus
```

Algorithm 3 Detecting composite knots on a chain

INPUT: a doubly linked list of beads after smoothing *atoms*
OUTPUT: a list of disconnected sub-lists of beads *subchains*

```
edges is a list of beads preceding any edge that separates subentanglements
for each B_i in atoms do
    midpoint = avg(B_i, B_{i+1})
    if smooth(atoms) is the same as smooth(atoms to midpoint) + smooth(atoms from midpoint) then
        add B_i to edges
end for
for each B_i in atoms do
    if B_i...B_{i+k} for k >= 4 not in edges then
        add list B_i...B_{i+k} to subchains
        go to loop index corresponding to B_{i+k+1}
    else
        go to loop index corresponding to B_{i+1}
end for
```

2. (moving a loop from over/under another):
   - if two crossings are consecutive twice (along both values, and consecutive values are both/neither marked as going on top) they can be removed (Figure 2B)
   - if the chain has the same overhandedness in two consecutive (along one value) crossings, they can possibly be replaced by one new crossing – this is verified by trying to find a second value (first being one of those consecutive) that would give a valid DT code – if no such value can be found, no simplification is made (Figure 2D);
3. (moving a segment completely over/under another crossing): this is the only move implemented which does not explicitly simplify the code, however it is attempted when no further simplification can be made (on condition that it will give a code not yet encountered).

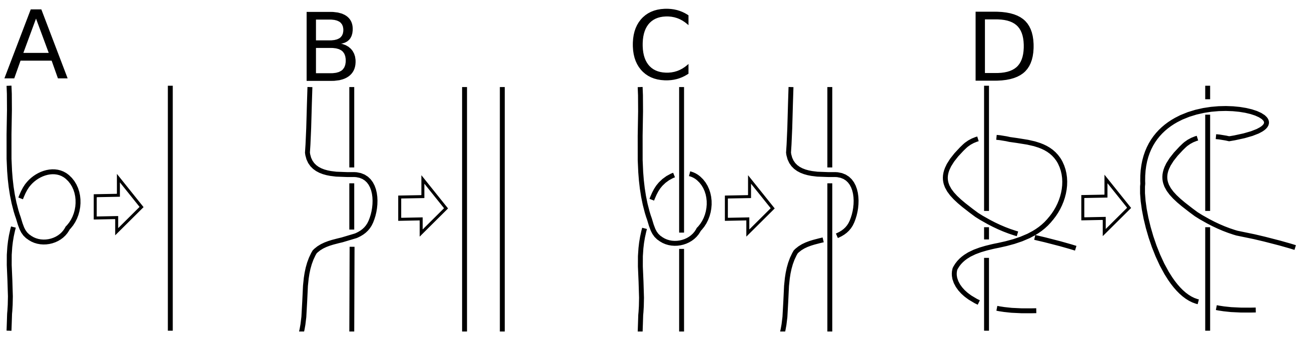

Fig. 2. Visualization of code simplifying moves implemented. A: based on Reidemeister move Type I. B: based on Reidemeister move Type II. C,D: based on a sequence of Reidemeister moves – Type III (twice for D), Type I, Type II.

When all possible simplifications have been exhausted, the number of crossings left should indicate the minimal number of crossings for the knot type present. Shortest DT code is then the sequence of even numbers from crossings sorted according to their odd values. If less than six crossings remain, this code is treated as final, and the corresponding Alexander-Briggs notation (found in a knot table) is returned as the final knot type. Starting from the six crossings, the knot can be actually a composite of simpler knots (e.g. two trefoil knots). When the DT code can be broken into permutations of consecutive sequences, each of them indicates a prime knot within the overall composite one (this complements the sub-chain separation during the smoothing). Each of those sub-knots is then indicated separately.

One of the characteristics of the DT code is that it is not unique for a generic closed curve – a given knot type can have multiple notations based on the starting point of numbering. This is not the case for open-chained knots, as both the starting point, and direction, of numbering are imposed by the protein chain. As knot tables contain only the lexicographically minimal DT code, we calculate also this code for proper translation to AB notation. If a given code doesn't appear in the tabulation, it is returned as a X_0, where X is the number of crossings in the shortest code found.

Our Alexander-Briggs notation does not include chirality of the knot, so to ensure that mirror image knots can be differentiated we the DT code returned contains also crossing orientation (as described by skein relations (Alexander and Briggs, 1926)), where if reoriented so that both crossing lines point up, "+" indicates that the one going left-to-right is on top, and "-" – that the one right-to-left.

Both space and time complexity of this algorithm are linear to the number of crossings – values pairs are kept in a list, which in each step of simplification is either shortened, or kept at the same length (and then it cannot go to a previously visited configuration).

3 Validation of results against KnotProt 2.0

To validate topology detection by knot_pull, it was ran against the KnotProt 2.0 (Dabrowski-Tumanski *et al.*, 2018) database, which is hand curated. Separate lists of chains assigned as knotted, slipknotted (full chain is unknotted, one or more sub-chains are knotted) and trivial protein chains were created. In case of structures reported by KnotProt 2.0 as trivial a random subset of 65 000 structures out of over 200 000 available was selected. All results which did not agree between knot_pull and KnotProt 2.0 are listed in Table 1.

Table 1. Protein chain which were found by knot_pull to contain different topology than reported by KnotProt 2.0

PDB Id	Chain	Knot type (KnotProt 2.0)	Knot type (knot_pull)	Note
5nfj	A	0_1 (unknot)	3_1	
5ush	A	0_1 (unknot)	3_1	
4r70	A	0_1 (unknot)	4_1	
4wr7	A	0_1 (unknot)	3_1	
5l6t	A	0_1 (unknot)	3_1	
5hya	A	0_1 (unknot)	3_1	
4coq	A	0_1 (unknot)	3_1	very shallow knot
1oq5	A	0_1 (slipknot: 3_1)	3_1	
5e5u	A	0_1 (slipknot: 3_1)	3_1	
3f7u	A	0_1 (slipknot: 3_1)	3_1	

References

Alexander, J. W. and Briggs, G. B. (1926). On types of knotted curves. *Annals of Mathematics*, pages 562–586.

Dabrowski-Tumanski, P. *et al.* (2018). Knotprot 2.0: a database of proteins with knots and other entangled structures. *Nucleic acids research*, **47**(D1), D367–D375.

Dowker, C. H. and Thistlethwaite, M. B. (1983). Classification of knot projections. *Topol. Appl.*, **16**(1), 19–31.

Koniaris, K. and Muthukumar, M. (1991). Self-entanglement in ring polymers. *J. Chem. Phys.*, **95**(4), 2873–2881.

Reidemeister, K. (1927). Elementare begründung der knotentheorie. In *Abhandlungen aus dem Mathematischen Seminar der Universität Hamburg*, volume 5, pages 24–32. Springer.
